# Photoinduced Glycerol Oxidation over Plasmonic Au and AuM (M = Pt, Pd and Bi) Nanoparticle-Decorated TiO_2_ Photocatalysts

**DOI:** 10.3390/nano8040269

**Published:** 2018-04-23

**Authors:** Trin Jedsukontorn, Nagahiro Saito, Mali Hunsom

**Affiliations:** 1 Fuels Research Center, Department of Chemical Technology, Faculty of Science, Chulalongkorn University, Bangkok 10330, Thailand; Trinatabo_jed@hotmail.com; 2 Graduate School of Engineering & Green Mobility Collaborative Research Center, Nagoya University, Nagoya 464-8603, Japan; hiro@sp.material.nagoya-u.ac.jp; 3 Center of Excellence on Petrochemical and Materials Technology (PETRO-MAT), Chulalongkorn University, Bangkok 10330, Thailand; 4 Associate Fellow of Royal Society of Thailand (AFRST), Sanam Suea Pa, Dusit, Bangkok 10300, Thailand

**Keywords:** glycerol conversion, gold nanoparticles, metal decorated TiO_2_, bimetallic nanoparticles, photocatalytic oxidation

## Abstract

In this study, sol-immobilization was used to prepare gold nanoparticle (Au NP)-decorated titanium dioxide (TiO_2_) photocatalysts at different Au weight % (wt. %) loading (Au*_x_*/TiO_2_, where *x* is the Au wt. %) and Au–M NP-decorated TiO_2_ photocatalysts (Au_3_M_3_/TiO_2_), where M is bismuth (Bi), platinum (Pt) or palladium (Pd) at 3 wt. %. The Au*_x_*/TiO_2_ photocatalysts exhibited a stronger visible light absorption than the parent TiO_2_ due to the localized surface plasmon resonance effect. Increasing the Au content from 1 wt. % to 7 wt. % led to increased visible light absorption due to the increasing presence of defective structures that were capable of enhancing the photocatalytic activity of the as-prepared catalyst. The addition of Pt and Pd coupled with the Au_3_/TiO_2_ to form Au_3_M_3_/TiO_2_ improved the photocatalytic activity of the Au_3_/TiO_2_ photocatalyst by maximizing their light-absorption property. The Au_3_/TiO_2_, Au_3_Pt_3_/TiO_2_ and Au_3_Pd_3_/TiO_2_ photocatalysts promoted the formation of glyceraldehyde from glycerol as the principle product, while Au_3_Bi_3_/TiO_2_ facilitated glycolaldehyde formation as the major product. Among all the prepared photocatalysts, Au_3_Pd_3_/TiO_2_ exhibited the highest photocatalytic activity with a 98.75% glycerol conversion at 24 h of reaction time.

## 1. Introduction

Biodiesel has recently become a globally attractive renewable biomass energy source. The increasing production of biodiesel has led to glycerol, the inevitable by-product from the biodiesel production process, becoming oversupplied in the market. To capitalize on the economic aspect of biodiesel production, it is essential to contrive a process for using glycerol in more valuable products. Due to the highly functionalized chemical molecule of glycerol, it is a potential bio-platform molecule that can be converted into viable chemicals and intermediate products for the chemical industry through a range of catalytic routes, such as fermentation [1], dehydrogenation [2], dehydration [3], etherification [4], esterification [5] and oxidation [6]. Consequently, the valorization of biodiesel-derived glycerol has gained much attention for increasing the proficiency and economic viability of the use of biomass resources. Among a large number of effective processes for glycerol transformation, aerobic oxidation is one of the extensively studied routes, since it can promote the generation of high-value fine chemicals and useful intermediates in organic synthesis, such as glycolic acid (GCOA), glyceric acid (GCA), glyceraldehyde (GCD), and dihydroxyacetone (DHA) [6,7]. However, the major hindrance in the glycerol oxidation process at a commercial scale is its requirement for an alkaline condition, high operating temperature and pressure, and the deactivation of the utilized catalyst. Thus, there is an urgent need to find a new, green process to address the increasing demands of sustainable and clean-energy technologies. In the last two decades, photocatalytic technology has been regarded as one of the most attractive processes due to its superior features, such as being environmentally benign, high efficiency, and operation at room temperature and atmospheric pressure. Many researchers have focused on this process, with versatile applications ranging from the field of wastewater and air remediation [8,9], carbon dioxide (CO_2_) reduction [10], clean hydrogen (H_2_) production [11] and the synthesis of high-value organic compounds [12], towards the development of an efficient solar-to-energy conversion [13].

A wide spectrum of semiconductors, such as TiO_2_, bismuth(III) oxide (Bi_2_O_3_), zinc oxide (ZnO), silicon carbide (SiC), tungsten(VI) oxide (WO_3_) and carbon nitride (C_3_N_4_), have been explored for their use in photocatalytic processes. Among all the commercial photocatalysts, TiO_2_ has the greatest appeal as a benchmark material in the fields of environmental remediation and solar fuel production, and still remains the most efficient and suitable material for light-energy harvesting. This is due to its many positive features, such as excellent photo-stability, high conductivity, low toxicity, low price and corrosion resistance. Despite these advantages, TiO_2_ is only optically active in the ultraviolet (UV) light region (~5% of the total solar spectrum), and together with the rapid recombination rate of photo-induced charge carriers. These are the main barriers that make TiO_2_ industrially less attractive. Several approaches to improve the photocatalytic performance of TiO_2_ nanostructures have been explored. The loading of TiO_2_ with a co-catalyst, such as metal nanoparticles (NPs) or secondary narrowed-bandgap semiconductors, to act as an electron-sink in order to boost the quantum yield is a promising effective approach. In addition, the decoration of metal NPs can also provide active sites for the reaction and alter the adsorption behavior of molecules by changing their polarizability, among other properties, leading to the preferential generation of the desired value-added products [14]. Various plasmonic noble metal NP-decorated TiO_2_ nanostructures that enhance the generation and migration of hot electrons from the metal to TiO_2_ have been widely reported [15,16,17]. Among all the decorated metal co-catalysts, Au has been found to be the most active metal, which is attributed to its noble nature, i.e., visible light active arising from the collective coherent oscillation of surface electrons through localized surface plasmon resonance (LSPR) [18,19,20,21,22], its low susceptibility to oxygen, high chemical stability [23] and effective charge transfer from TiO_2_ to it and/or *vice versa* [24]. The synthesis of Au NPs has become of great interest in recent decades. The most impressive technique for AuNP synthesis is the two-phase reduction method developed by Brust and co-workers [25,26]. The breakthrough for using Au NPs as a heterogenous catalyst has occurred since Haruta and Hutching provided the significant observations that Au NPs was very active for CO oxidation and ethylene hydrochlorination [27,28]. Apart from that, to date many reports have pointed out that Au NPs are also active in a range of catalytic routes such as glycerol conversion [7,29,30], water splitting [20,31], H_2_ generation [21,32], the photo-oxidation of organic substrates [22,33,34,35] and the photo-reduction of organic compounds [36,37].

Besides the monometallic Au, the combination of Au NPs with other metals can enable an enhanced global reaction system and promote the desired reaction pathway depending on their structural and/or electronic properties at the nanoscale level. Bimetallic Au-based NPs have been studied in a variety of reactions, including solvent-free benzyl alcohol oxidation to benzaldehyde [38]; selective oxidation of methanol to methyl formate [39]; photocatalytic H_2_ production from ethanol reforming over core–shell Au-palladium (Pd) promoters [40] and over bimetallic Au-platinum (Pt) NPs [41]; selective oxidation of furfural to furoic acid [42]; selective oxidation of 1,2-propanediol to lactic acid [43]; selective oxidation of ethanol to acetaldehyde [44]; and the degradation of ciprofloxacin [45]. Apart from these reactions, glycerol oxidation in a liquid phase system has also been intensively investigated over a bimetallic Au-based system [46,47,48,49]. The synergism between Au and a coupled metal (e.g., Pt or Pd) can boost up the glycerol conversion and tune the selectivity of oxidation products. Moreover, the synthesis procedure and particle size of as-prepared bimetallic Au-based NPs also significantly affected the product distribution and route of glycerol transformation [47,50,51].

As far as we know, there have been only a few research reports on the photocatalytic oxidation of glycerol over metal-decorated TiO_2_. Panagiotopoulou et al. [52] prepared Pt impregnated on TiO_2_ and utilized the prepared Pt/TiO_2_ for the photo-reforming and photo-oxidation of glycerol. The Pt/TiO_2_ photocatalyst exhibited a more efficient reaction rate for glycerol oxidation than bare TiO_2_, which was mainly due to the increased separation of the charge carriers and the elevation of the rate-limiting cathodic half reaction (oxygen (O_2_) reduction) in the glycerol photo-oxidation. Likewise, the addition of any of Bi, Au, Pt or Pd on TiO_2_ was reported in our previous work to further enhance the rate of glycerol photo-oxidation, where Au-decorated TiO_2_ exhibited the best performance [53]. However, the photocatalytic conversion of glycerol over the surface of metals and metal oxides to the desired products still requires a thorough comprehension of the surface chemistry and reaction mechanism. The bias towards certain reaction pathways can be modulated by varying the reaction conditions (light, pressure, temperature, etc.), reaction media (water with O_2_ or hydrogen peroxide) and the nature of the catalyst surface (i.e., monometal, bimetal and/or metal oxide). Thus, in this work, we investigated the photocatalytic oxidation of glycerol over Au-based mono- and bimetallic NP-decorated TiO_2_. The aim was to explore the individual as well as the combined effects of Au, Au-Bi, Au-Pt and Au-Pd on the photocatalytic performance of TiO_2_ for the photocatalytic oxidation of glycerol. The Bi, Pt and Pd NPs were selected to combine with Au because Bi prefers to react with the secondary hydroxyl group of glycerol to form DHA while the Pt and Pd can promote the activity of glycerol oxidation [53,54,55,56].

## 2. Experiment

### 2.1. Preparation of the Au/TiO_2_ and AuM/TiO_2_ Photocatalysts

Both mono- and bimetallic NP-decorated TiO_2_ photocatalysts were prepared by sol-immobilization, which has been previously reported to be an outstanding preparation method [51,57]. Commercial TiO_2_ powder (Sigma Aldrich, St. Louis, MO, USA) containing ≥99% anatase phase structure was used as the support semiconductor material. The Au NP-decorated TiO_2_ (Au/TiO_2_) at different Au contents from 1 wt. % to 7 wt. % were prepared using sodium borohydride (NaBH_4_; Loba Chemie, Mumbai, India) as a reducing agent. In brief, for the 1 wt. % Au NP-decorated TiO_2_ (Au_1_/TiO_2_), 40.8 mg of HAuCl_4_·3H_2_O (Sigma Aldrich) was dissolved in deionized (DI) water. Then, 2 wt. % polyvinyl alcohol (PVA; [-CH_2_CHOH-]*_n_*, 99% hydrolyzed, Sigma Aldrich) was added to a 1:10 molar ratio of PVA: HAuCl_4_·3H_2_O with stirring at a constant rate of 300 rpm in order to stabilize the metal dispersion on the support and prevent agglomeration. Next, 1.98 g of TiO_2_ (Anatase, Sigma Aldrich) was added to the prepared solution and then excess NaBH_4_ was slowly added to the colloidal solution. The reaction was held for 24 h at atmospheric pressure and temperature to obtain the complete sol immobilization. The obtained mixture was filtered and washed thoroughly several times with DI water until no chloride ions remained, which was detected in the filtrate solution by the silver nitrate test. Finally, the obtained solid was dried at 110 °C in a hot air oven overnight and, subsequently, the organic scaffold residue was removed and the catalyst was activated by heat treatment under a N_2_ flow at 350 °C for 3 h followed by a H_2_ flow at 350 °C for 3 h, respectively, to yield the ready-to-use Au_1_/TiO_2_. A similar procedure was repeated for the 3, 5 and 7 wt. % Au NP-decorated TiO_2_, except using 122.45, 204.06 and 285.72 mg of HAuCl_4_·3H_2_O instead of 40.8 mg to yield the Au_3_/TiO_2_, Au_5_/TiO_2_ and Au_7_/TiO_2_ photocatalysts, respectively.

To prepare the Au-based bimetallic NP-decorated TiO_2_ photocatalysts with a 3 wt. % loading of both Au and the selected metal NPs (Au_3_M_3_/TiO_2_), three types of second metal (Bi, Pt, and Pd) were separately incorporated with the Au on TiO_2_ to yield the Au_3_Bi_3_/TiO_2_, Au_3_Pt_3_/TiO_2_ and Au_3_Pd_3_/TiO_2_ photocatalysts, respectively, via the same method. For the Au_3_Bi_3_/TiO_2_ photocatalyst, 122.45 mg of HAuCl_4_·3H_2_O was dissolved in 10 mL of DI water, while 90.9 mg of bismuth(III) chloride (BiCl_3_; Sigma Aldrich) was dissolved in 10 mL of 0.2 M of hydrochloric acid (HCl; 37% purity, Sigma Aldrich). Both solutions were mixed together and added to 10.5 mL of 2 wt. % PVA with stirring at constant rate of 300 rpm in order to stabilize the metal dispersion on the support and prevent agglomeration. Then, 1.88 g of TiO_2_ was added to the prepared solution and subsequently reduced by the slow addition of NaBH_4_ to get the colloid solution. The reaction was held for 24 h to obtain complete sol immobilization. Then, the obtained mixture was filtered and washed thoroughly with DI water until no chloride ions. Finally, the obtained solid was dried in an oven and subsequently removed the organic scaffold residue and activated by heat treatment under a N_2_ flow followed by a H_2_ flow as above to yield the ready-to-use Au_3_Bi_3_/TiO_2_. Similar procedures were repeated for the synthesis of the Au_3_Pt_3_/TiO_2_ and Au_3_Pd_3_/TiO_2_ photocatalysts except using 159.6 mg of chloroplatinic acid hexahydrate (H_2_PtCl_6_·6H_2_O; Sigma Aldrich) dissolved in 10 mL of DI water and 100 mg of palladium (II) chloride (PdCl_2_; Sigma Aldrich) solution dissolved in 10 mL of 0.2 M HCl instead of the BiCl_3_.

### 2.2. Characterization of the as-Prepared Au_x_/TiO_2_ and Au_3_M_3_/TiO_2_ Photocatalysts

The diffractogram peaks of all prepared photocatalysts were first characterized by X-ray diffraction (XRD) using a D8 Discover-Bruker AXS X-ray diffractometer (Billerica, MA, USA) equipped with Cu K*α* in order to evaluate the crystal and phase structure. The X-ray diffractometer was operated at 40 mA and 40 kV with acquiring step of 0.02° between angles of 10–80 degrees. The diffractogram peaks were identified using the JCPDS database. The loading content of the decorated metal on the TiO_2_ surface was examined by scanning electron microscopy (SEM; JSM-6610LV) (Peabody, MA, USA) equipped with energy-dispersive X-ray spectrometry (EDS) to perform elemental analysis at the atomic resolution. The dispersion of decorated-metals and particle size distribution were observed by transmission electron microscopy (TEM; JEOL-2100Plus, Tokyo, Japan) at an electron acceleration of 200 kV. Elemental line scan and mapping of the decorated metal were also observed on a JEOL 2100 Plus equipped with a Bruker EDS using a nominal electron beam size of 1 nm. The average metal particle sizes and their distribution were calculated by averaging not less than 100 particles randomly distributed in the TEM images. The diffuse reflectance spectra were monitored via UV-visible near infrared spectrometry (UV-Vis-NIR; Perkin Elmer, Lambda 950, Waltham, MA, USA) over a wavelength of 320–820 nm. The elemental oxidation states of all the prepared photocatalysts were assessed by X-ray photoelectron spectroscopy (XPS; PHI 5000 VersaProbeII) (ULVAC-PHI, Inc., Kanagawa, Japan) with a monochromatized Al K*α* source (h*ν* = 1486.6 eV). Accurate binding energies (±0.1 eV) were established with respect to the position of the adventitious carbon C1s peak at 284.8 eV. The spectra deconvolution was performed using the XPSPEAK41 software package (Kratos Analytical Ltd., Manchester, UK).

### 2.3. Photocatalytic Activity Test

The photocatalytic activity of all the prepared photocatalysts was tested via the photocatalytic oxidation of glycerol at room temperature and ambient pressure using a 120 W high-pressure mercury lamp (RUV 533 BC, Holland, The Netherlands) at light intensity 4.7 mW/cm^2^. The photoreactor was placed in the middle of a UV-protected box (0.68 m × 0.68 m × 0.78 m) containing 100 mL of 0.3 M glycerol (QReC). The solution was agitated at 300 rpm to achieve complete mixing. Oxygen was supplied continuously into the reactor at a constant feed rate of 200 mL/min. Prior to light illumination, the selected photocatalyst was suspended in the glycerol solution in a dark room for 30 min to reach an adsorption equilibrium. As the experiment progressed, 2 mL of sample was collected and then centrifuged on a KUBOTA KC-25 digital laboratory centrifuge (KUBOTA, Tokyo, Japan) to separate the solid catalyst from the aqueous product. The variation in the glycerol concentration and the generation of all monitored compounds, including GCD, DHA, hydroxypyruvic acid (HPA), GCOA, formaldehyde (FMD) and glycolaldehyde (GCAD), were analyzed by high-performance liquid chromatography with a RID-10A refractive index detector (Shimadzu, LC-10 ADVP, Kyoto, Japan). The stationary phase was an Aminex HPX-87H ion-exclusion column (300 × 7.8 mm), and the mobile phase was a 70:30 (*v*/*v*) water: acetonitrile solution in 0.5 mM H_2_SO_4_ at a constant flow rate of 0.4 mL/min. Standard solutions of glycerol, GCD (Sigma Aldrich), DHA (Merck, Kenilworth, NJ, USA), HPA (Sigma Aldrich), GCA (Ajax Finechem, Taren Point, Australia), FMD (Merck), GCOA (Ajax Finechem) and GCAD (Sigma Aldrich) were run to identify the retention times and determine the relationships between the peak area and concentration. The conversion of glycerol (*X*) and yield (*Y*) of each selected product of the photocatalytic oxidation were calculated according to Equations (1) and (2), respectively:

(1) $$X(\%)=\frac{\mathrm{Amount}\;\mathrm{of}\;\mathrm{glycerol}\;\mathrm{converted}\;(C-\mathrm{based}\;\mathrm{mole})}{\mathrm{Total}\;\mathrm{amount}\;\mathrm{of}\;\mathrm{glycerol}\;\mathrm{in}\;\mathrm{reactant}\;(C-\mathrm{based}\;\mathrm{mole})}\times 100$$

(2) $$Y(\%)=\frac{\mathrm{Amount}\;\mathrm{of}\;\mathrm{glycerol}\;\mathrm{converted}\;\mathrm{to}\;\mathrm{product}\;j\;(C-\mathrm{based}\;\mathrm{mole})}{\mathrm{Total}\;\mathrm{amount}\;\mathrm{of}\;\mathrm{glycerol}\;\mathrm{in}\;\mathrm{reactant}\;(C-\mathrm{based}\;\mathrm{mole})}\times 100$$

## 3. Results and Discussion

### 3.1. Au/TiO_2_ Photocatalysts

#### 3.1.1. Photocatalyst Morphology

**Figure 1:**
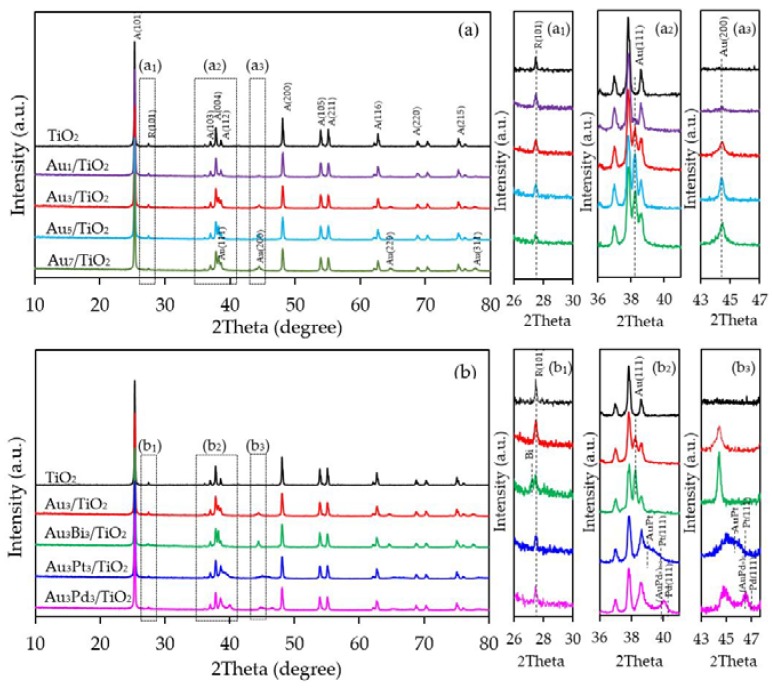
Representative X-ray diffraction (XRD) patterns of the (**a**) Au*_x_*/TiO_2_ and (**b**) Au_3_M_3_/TiO_2_ photocatalysts.

Representative XRD patterns of the as-synthesized Au*_x_*/TiO_2_ photocatalysts with different metal loading contents are displayed in Figure 1a. All photocatalysts exhibited the main diffraction peaks of TiO_2_ in the anatase phase at a 2*θ* of 25.3°, 37.0°, 37.9°, 38.6°, 48.1°, 53.9°, 55.1°, 62.7°, 68.8°, 70.3° and 75.1°, corresponding to the (101), (103), (004), (112), (200), (105), (221), (204), (116), (220) and (215) crystal planes, respectively (JCPDS No. 21-1272), with an additional rutile peak in trivial content at 27.5° corresponding to the (101) crystal plane (JCPDS No. 04-0802) (Figure 1a_1_). In addition, the diffraction peaks of Au NPs appeared at about 38.2°, 44.4°, 64.6° and 77.6°, assigning to the face-centered cubic structure of Au with (111), (200), (220) and (311) planes (JCPDS No. 002-1095), indicating the presence of metallic Au in the as-synthesized Au*_x_*/TiO_2_ photocatalysts. The intensity of the Au peaks increased as the content of Au decorated on the TiO_2_ surface increased, but without a shift in the peak position (Figure 1a_2_,a_3_).

**Figure 2:**
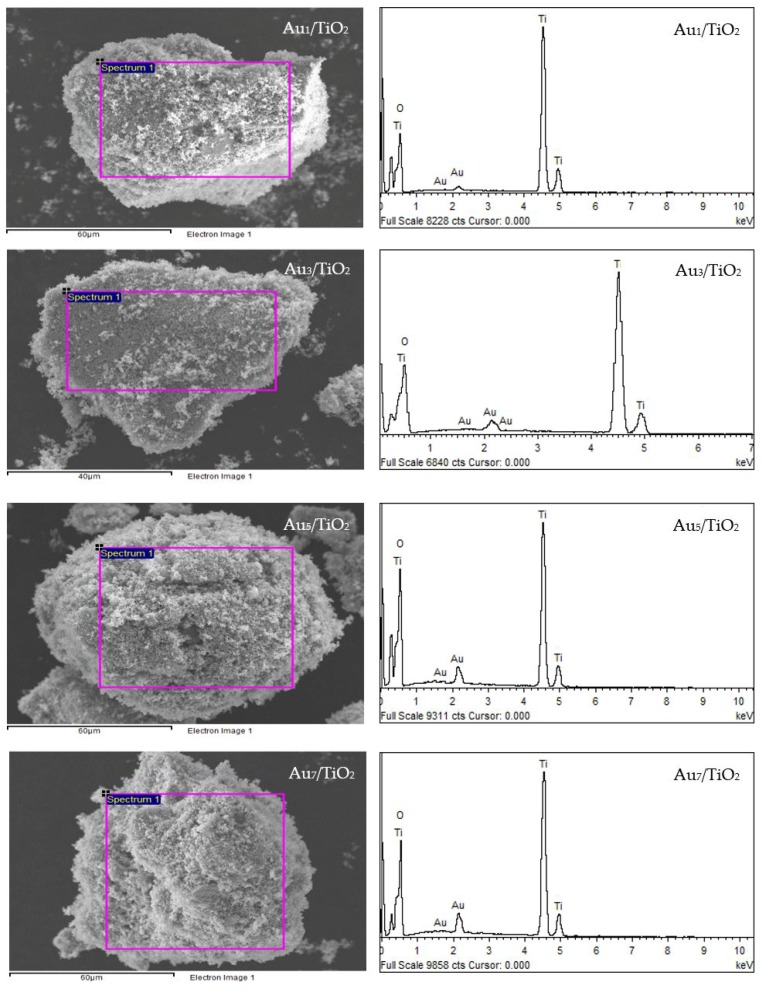
(**Left**) Representative SEM images of the different Au*_x_*/TiO_2_ photocatalysts and (**Right**) the EDS elemental analysis of the corresponding area shown in the rectangle on the SEM image.

**Figure 3:**
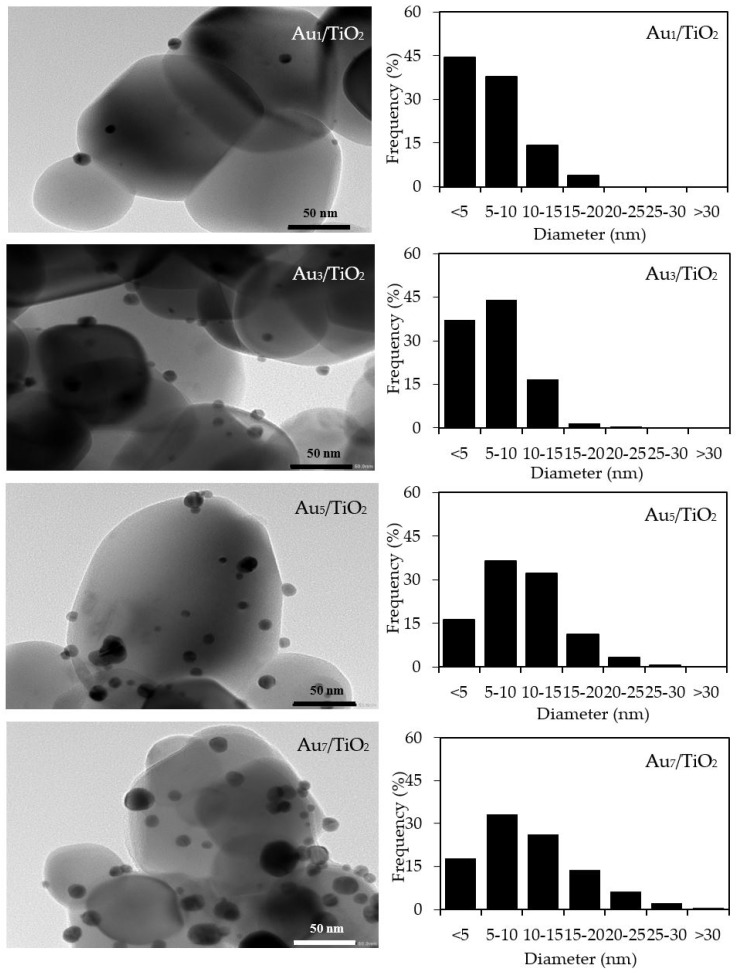
(**Left**) Representative TEM images of the different Au*_x_*/TiO_2_ photocatalysts, and (**Right**) the derived Au nanoparticle (NP) size distribution on the surface of the TiO_2_.

The qualitative presence of Au in all the Au*_x_*/TiO_2_ photocatalysts was also confirmed by SEM–EDS analysis (Figure 2). Quantitatively, as summarized in Table 1, the Au content in each Au*_x_*/TiO_2_ photocatalyst was close to the set value. The TEM images of the as-prepared photocatalysts with the derived Au NPs size distribution are displayed in Figure 3. They exhibited well-dispersed decorated Au NPs on the TiO_2_ surface with a narrow size distribution. The average size of Au NPs increased slightly as the Au content increased, suggesting an agglomeration of Au NPs in the presence of a high Au loading content.

**Figure 4:**
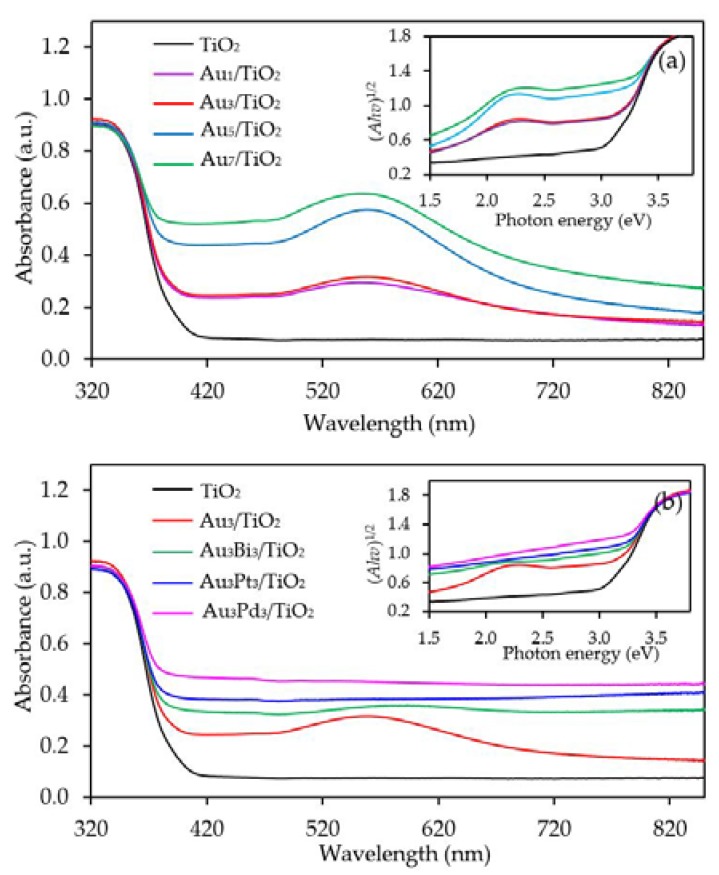
Representative ultra violet-visible (UV-Vis) spectra of the TiO_2_ and different (**a**) Au*_x_*/TiO_2_ and (**b**) Au_3_M_3_/TiO_2_ photocatalysts with (insert) the respective Tauc’s plots.

Figure 4a exhibits the UV-vis absorption spectra of the Au*_x_*/TiO_2_ photocatalysts with different Au contents. The parent TiO_2_ exhibited the main intense UV absorption band at a wavelength of less than 400 nm with no absorption band in the visible light region or at a wavelength of greater than 400 nm. This was mainly due to its large bandgap energy that enabled the electron transfer from the filled valence O 2p orbitals of the valence band to the vacant Ti 3d orbitals of the conduction band only by the excitation of the photon energy from the UV light region. However, all the Au*_x_*/TiO_2_ photocatalysts exhibited an enhanced visible light absorption with an intense broad absorption band centered at wavelength around 540 nm. The peak intensity increased as the Au content increased from 1.0 wt. % to 7.0 wt. %. The presence of the visible light absorption of all Au*_x_*/TiO_2_ photocatalysts might be caused by the LSPR effect of the decorated Au NPs that can absorb visible light through the polarization and oscillation of the conduction electrons in the metal structure [58]. However, as reported previously, the LSPR band of well-dispersed spherical Au nanocrystals is generally sharp and appears at a wavelength of 520 nm [59]. The absorption center of all the prepared Au*_x_*/TiO_2_ photocatalysts in this study deviated from the mentioned wavelength (520 nm) with a broad tail extending towards longer wavelengths, which might be attributed to the anisotropy of the trigonal/prismatic shape (non-spherical) of the Au nanocrystals [60]. The bandgap values of all samples were determined from a Tauc’s plot (inset of Figure 4a), and are listed in Table 1. The addition of Au NPs at 1 and 3 wt. % affected insignificantly the bandgap energy of the parent TiO_2_. In that, both contents decreased the bandgap value of TiO_2_ from 3.02 eV to 3.0 eV, probably because both low Au contents can induce only a small formation of Ti^3+^ defect structure which cannot intensely alter the major electronic state position of the O2p valence orbital as well as the localized band bending of the O2p valence band edge maximum. However, addition of Au NPs at loading of 5 and 7 wt. % importantly decreased the band-gap value of TiO_2_ to 2.94 and 2.80 eV, respectively. This indicated that the presence of a high defective TiO_2_ structure in the presence of high Au content (5 and 7 wt. %) can alter the major electronic state of the valence band and conduction band position.

**Figure 5:**
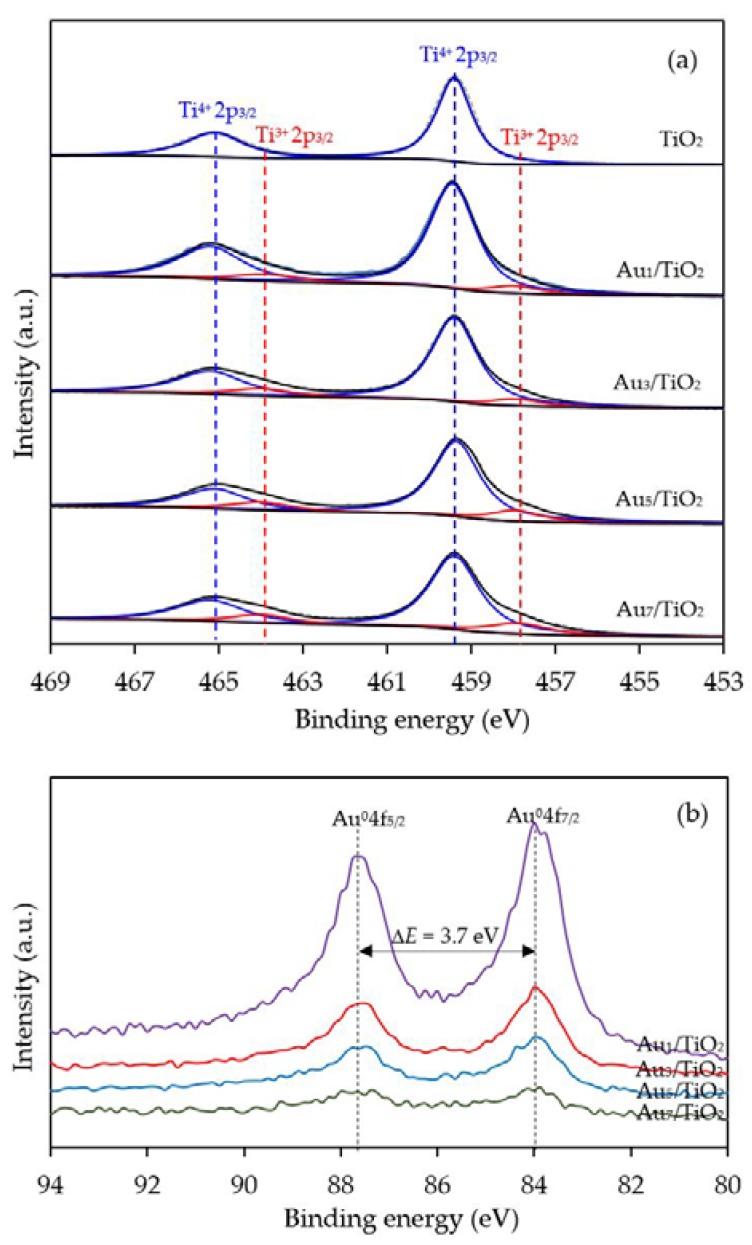
Representative c spectra of the (**a**) Ti2p and (**b**) Au4f of the TiO_2_ and different Au*_x_*/TiO_2_ photocatalysts.

To confirm the formation of defective structure of TiO_2_ in the presence of Au NPs, XPS analysis with a high resolution (HR) Ti2p state was employed. As demonstrated in Figure 5a, two symmetrical peaks at 459.4 and 465.1 eV, assigned to Ti2p_3/2_ and Ti2p_1/2_, were exhibited in the Ti2p core-level spectra of all the prepared Au*_x_*/TiO_2_ photocatalysts. The different binding energy of the observed spin-orbit splitting between the Ti2p_3/2_ and Ti2p_1/2_ was around 5.7 eV, in accordance with the typical value of Ti^4+^ sites coordinated to oxygen atoms in TiO_2_ [61]. Interestingly, two additional tailored peaks were observed at a lower binding energy of around 457.9 and 464.0 eV, which can be ascribed to the existence of Ti^3+^ species. The generation of Ti^3+^ could have originated from the incorporation of the decorated-metal on the TiO_2_ under the H_2_ treatment [62,63]. This is because the decorated metal, Au in this case, can induce the generation of oxygen vacancies that are subsequently essential to form the Ti^3+^-O^−^-Ti^4+^ defect structures [64]. After deconvolution, the relative content of Ti^3+^/Ti^4+^ in all the Au*_x_*/TiO_2_ photocatalysts was obtained, and is summarized in Table 1. The Ti^3+^/Ti^4+^ ratio increased as the Au content increased, becoming almost two-fold higher in Au_7_/TiO_2_ than in Au_1_/TiO_2_, suggesting that the decoration of TiO_2_ with a high Au content induced a high content of Ti^3+^ defective structures in the TiO_2_.

With respect to the valence state of the decorated metal, the Au 4f level spectra were also examined. All the prepared photocatalysts displayed the main Au 4f_7/2_ and Au 4f_5/2_ peaks at a binding energy of about 84.0 and 87.7 eV with a spin-orbit splitting of 3.7 eV (Figure 5b), which are in excellent agreement with the values of the monometallic Au^0^ state [65]. Therefore, the Au NPs existed totally in metallic form on the surface of the as-prepared Au*_x_*/TiO_2_ photocatalysts. No shift in the Au 4f binding energy was observed, suggesting that the addition of Au within the investigated weight content (1–7 wt. %) did not alter the chemical state of the decorated Au NPs on the TiO_2_.

#### 3.1.2. Photocatalytic Activity

**Figure 6:**
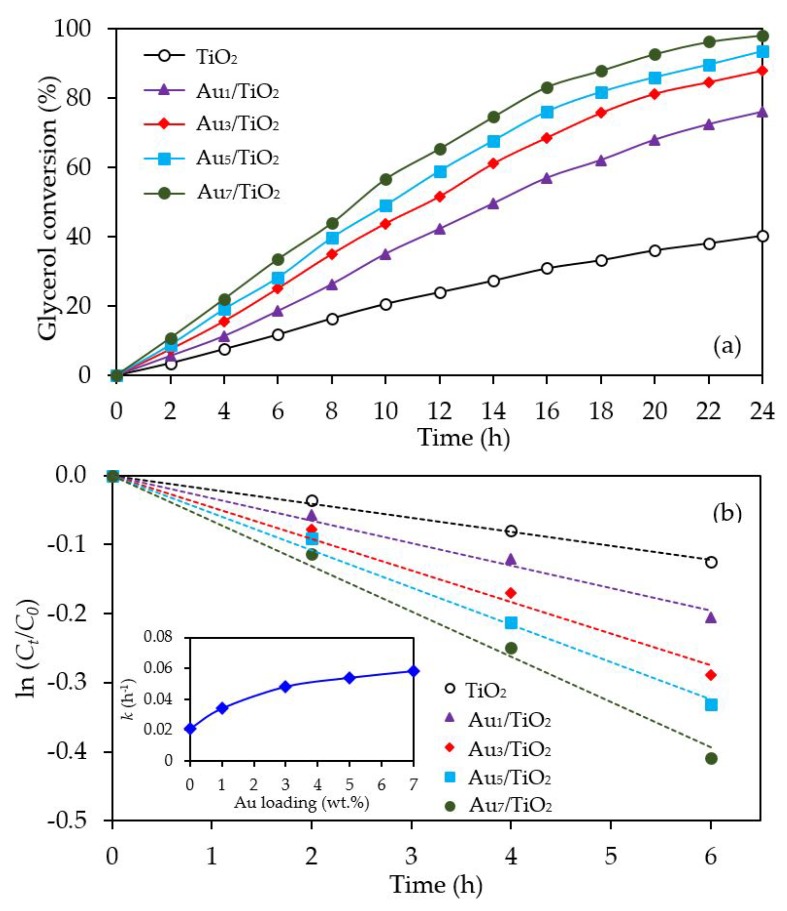
Representative (**a**) glycerol conversion and (**b**) plot of ln (*C_t_*/*C*_0_) as a function of time in the presence of the TiO_2_ or different Au*_x_*/TiO_2_ (*x* = 1–7) photocatalysts at a loading of 3.0 g/L, light intensity of 4.7 mW/cm^2^ and using O_2_ as the electron acceptor.

The glycerol conversion in the presence of the different Au*_x_*/TiO_2_ photocatalysts is summarized in Figure 6a. All the Au*_x_*/TiO_2_ photocatalysts significantly enhanced the glycerol conversion compared to the bare TiO_2_. The glycerol conversion increased as the Au content increased from 1 wt. % to 7 wt. %. Plotting the data with the pseudo first order reaction kinetic model (Figure 6b), where a plot of ln (*C_t_*/*C*_0_) versus *t* provides a straight line and allows the determination of the kinetic rate constant (*k*), gave a good fit with a determination coefficient (*R*^2^) of greater than 0.9879. Accordingly, the kinetic rate constant of glycerol conversion over the Au_1_/TiO_2_, Au_3_/TiO_2_, Au_5_/TiO_2_ and Au_7_/TiO_2_ photocatalysts were deduced to be 0.0340, 0.0480, 0.0537 and 0.0606 h^−1^, respectively, compared to 0.021 h^−1^ for TiO_2_ (inset of Figure 6b). The enhanced glycerol conversion over Au-decorated TiO_2_ could have arisen from the plasmon induction effect of Au NPs. In addition, the presence of Au NPs was able to serve as electron sinks to facilitate interfacial electron transfer, which could effectively reduce the recombination rate of photogenerated holes (*h*^+^) and electrons (*e*^−^) [66].

**Figure 7:**
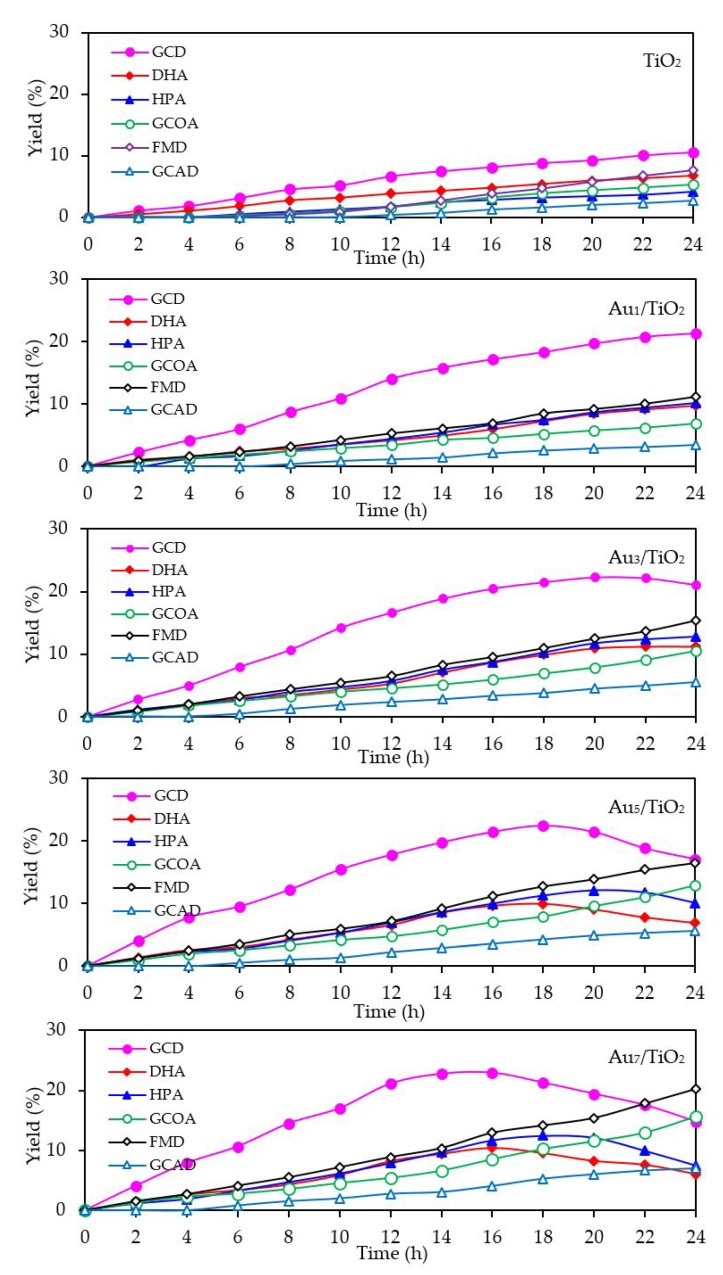
Yield of selected products obtained from the glycerol photocatalytic conversion via the TiO_2_ and different Au*_x_*/TiO_2_ photocatalysts at a loading of 3.0 g/L, light intensity of 4.7 mW/cm^2^ and using O_2_ as the electron acceptor.

With respect to the chemical species generated from the glycerol conversion via the photocatalytic oxidation process in the presence of the Au*_x_*/TiO_2_ photocatalysts, similar chemical species, including GCD, DHA, HPA, GCOA, FMD and GCAD, were generated via photocatalytic oxidation with the Au*_x_*/TiO_2_ photocatalysts as well as with the parent TiO_2_ (Figure 7), where GCD was the main product. However, the GCD yield as a function of reaction time varied with the different Au NP contents on the Au*_x_*/TiO_2_ photocatalysts. The yield of GCD increased continuously in the presence of 1 wt. % Au_1_/TiO_2_ from the initial time to the end of reaction time, but in the presence of a high Au content (Au ≥ 3 wt. %), the yield of GCD increased continuously to reach a maximum value of around 22% and then dropped slightly. The Au*_x_*/TiO_2_ photocatalysts with a high Au NP content enhanced the fast generation of GCD to a maximum level and then accelerated the conversion of GCD to other chemical species, as seen by the decreased GCD yield in the later reaction times. This suggested that the Au*_x_*/TiO_2_ photocatalysts with a higher Au content had a higher photocatalytic activity than those with a lower Au content. This was presumably due to their higher content of defective structures, which could promote the visible light absorption due to the narrowing of the electronic bandgap via the continuum interstate between the Ti3d states of the conduction band and the O2p states of the valence band [67]. A high quantity of absorbed photons can achieve a high level of photogenerated *h*^+^ and *e*^-^ on the TiO_2_ surface. Moreover, the function of the decorated Au NPs as an electron sink can also promote the lifetime of the photogenerated *h*^+^ and *e*^−^, and so enhanced the photocatalytic activity for glycerol conversion.

The mechanism of glycerol conversion to GCD, DHA, HPA, GCOA, FMD and GCAD has been proposed previously [12,68,69]. In brief, the photocatalytic oxidation of glycerol via TiO_2_ occurred mainly at the 1°-OH groups to form GCD as the principle product and partially occurred at the 2°-OH group to form DHA. The generated GCD can then be further oxidized with hydroxyl radicals (HO^•^) to form GCOA and FMD, while the DHA is then further oxidized with oxygen species (O_2_^•−^/^1^O_2_) to form HPA. The generation of a low GCAD content was probably caused by the adsorption of two molecules of generated FMD on two neighboring Ti^3+^ defective sites and their subsequent dimerization to form GCAD.

### 3.2. Au_3_M_3_/TiO_2_ Photocatalysts

According to the results obtained, to further explore the role of decorated bimetallic NPs and enhance the glycerol conversion efficiency and yield of product distribution, the Au_3_/TiO_2_ photocatalyst was selected to composite with other three metals (Bi, Pt and Pd) at the same loading of 3 wt. %.

#### 3.2.1. Photocatalyst Morphology

**Figure 8:**
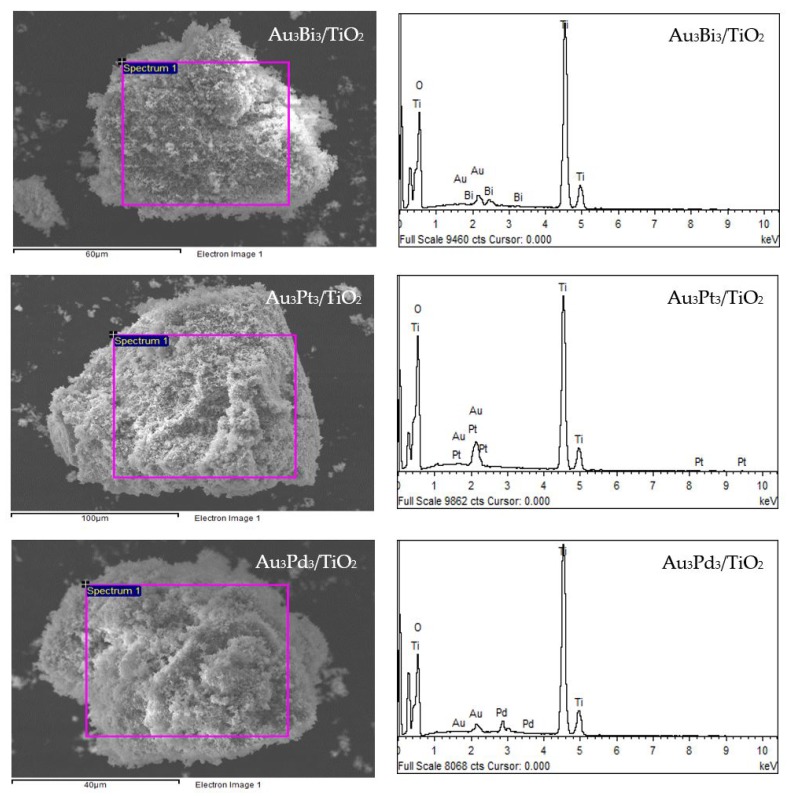
(**Left**) Representative SEM images of the different Au_3_M_3_/TiO_2_ photocatalysts and (**Right**) EDS elemental analysis of the corresponding area shown in the rectangle on the SEM image.

The XRD patterns of the prepared Au_3_M_3_/TiO_2_ photocatalysts are displayed in Figure 1b. All three exhibited the main diffraction peaks of TiO_2_ in the anatase phases, while the diffraction peak of Au NPs was still clearly observed in the XRD patterns of the Au_3_/TiO_2_ and Au_3_Bi_3_/TiO_2_ photocatalysts, but was not observed in the Au_3_Pt_3_/TiO_2_ and Au_3_Pd_3_/TiO_2_ photocatalysts (Figure 1b_1_). A clear diffraction peak of Bi in the rhombohedral lattice appeared separately at a 2*θ* of 27.0° (PDF No. 01-085-1329) (Figure 1b_1_), while no separate peak of Pt and Pd was clearly observed, probably due to their alloy formation with Au NPs. The track of AuPt and AuPd peaks was detected, as shown in Figure 1b_2_,b_3_. The presence of Au and the respective M in all three Au_3_M_3_/TiO_2_ photocatalysts was close to the set values (3 wt. % of both Au and M), as confirmed by the SEM–EDS analysis (Figure 8), and are listed in Table 1.

**Figure 9:**
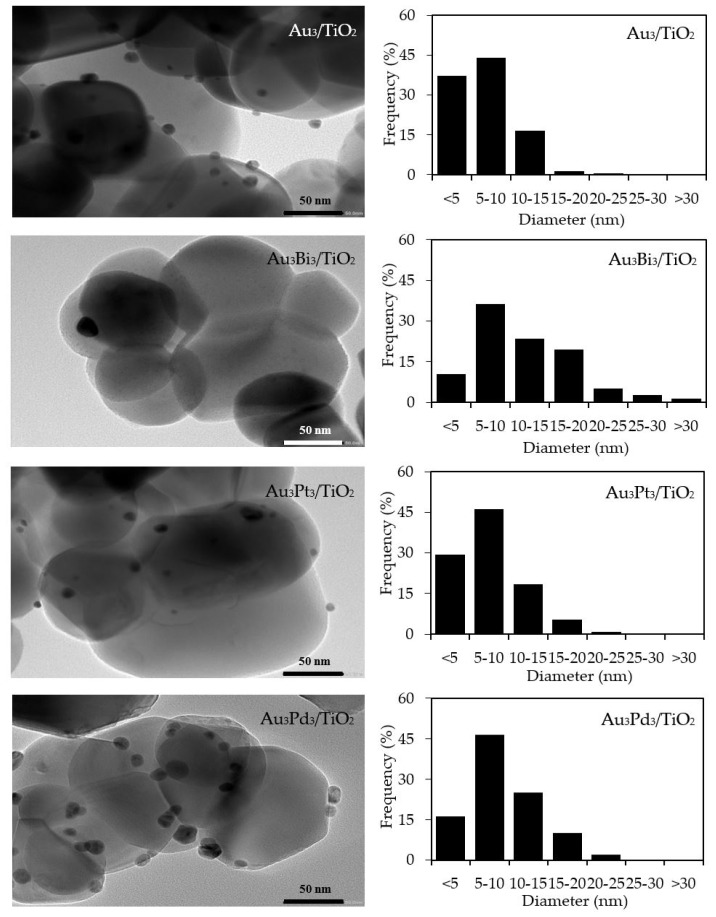
(**Left**) Representative TEM images of the Au_3_/TiO_2_ and different Au_3_M_3_/TiO_2_ photocatalysts and (**Right**) the derived respective AuM NP size distribution on the surface of the TiO_2_.

**Figure 10:**
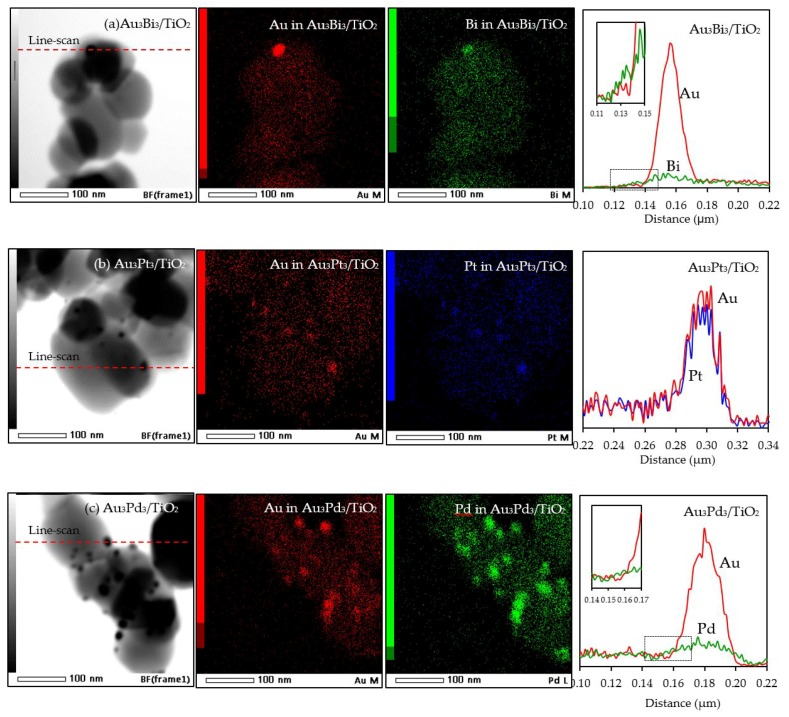
Representative TEM–EDS mapping images and line-scan profile of the different of Au_3_M_3_/TiO_2_ photocatalysts.

With respect to the particle size distribution, the TEM images (Figure 9) revealed a well-dispersed and narrow size distribution of the decorated AuM NPs on the TiO_2_ surface, with a slightly larger particle size than that in Au_3_/TiO_2_ (Table 1). This suggests that the addition of the second metal induced a slight agglomeration of the AuM NPs. To get insight into the morphology of the decorated metal NPs in the bimetallic catalysts, EDS line-scanning together with the elemental mapping analysis was performed. The compositional line scan of the Au_3_Bi_3_/TiO_2_ photocatalyst showed that the concentration of Au increased from the edge to the center of the metal particle, whereas the Bi distribution was almost constant from the edge to the center of the selected particle (Figure 10a), indicating a core–shell structure of the Bi–Au particle with Au as the core and Bi as the shell. The onset position of the Bi signal was around 0.13 μm, which was 0.008 μm lower than that of Au, indicating a 8 nm thickness of the dendritic Bi shell. A core–shell structure was also observed for the Au_3_Pd_3_/TiO_2_ photocatalyst, in which the high density of Au appeared at the center of the metal particle, while Pd was distributed uniformly from the edge to the center (Figure 10c), with the thickness of the dendritic Pd shell around the Au core being 7.5 nm. A different pattern of the line scan profile was observed for the Au_3_Pt_3_/TiO_2_ photocatalyst, in which the density of both Au and Pt NPs increased from the edge to the center of the selected particle (Figure 10b). This indicated that the AuPt ensembles existed as a solid solution of arbitrary composition or as an AuPt alloy form.

The capability of UV-vis absorption spectra of the Au_3_M_3_/TiO_2_ photocatalysts is shown in Figure 4b, where they all exhibited a high capability to absorb visible light at a wavelength of greater than 400 nm. The addition of Bi, Pt or Pd to the Au_3_/TiO_2_ attenuated an intense broad absorption band centered at around 540 nm of Au, probably due to the different dielectric function of the bimetals compared to the Au monometallic state [70]. Moreover, the strong interaction between the decorated Au–M can also influence the flattening of the Au LSPR band [71]. The bandgap values of the Au_3_Bi_3_/TiO_2_, Au_3_Pt_3_/TiO_2_ and Au_3_Pd_3_/TiO_2_ photocatalysts, as determined from a Tauc’s plot (inset of Figure 4b), were 2.85, 2.85 and 2.79 eV, respectively (Table 1), which suggested that the addition of the respective second metal caused the generation of the defective structure of TiO_2_. The formation of the Ti^3+^ defect structure likely induced an altered major electronic state of the valence band and conduction band, by which it formed a mid-gap state below the conduction band and also enabled the lengthening of the conduction band edge, resulting in the formation of the band tail structure and the narrowness of the bandgap energy [72].

**Figure 11:**
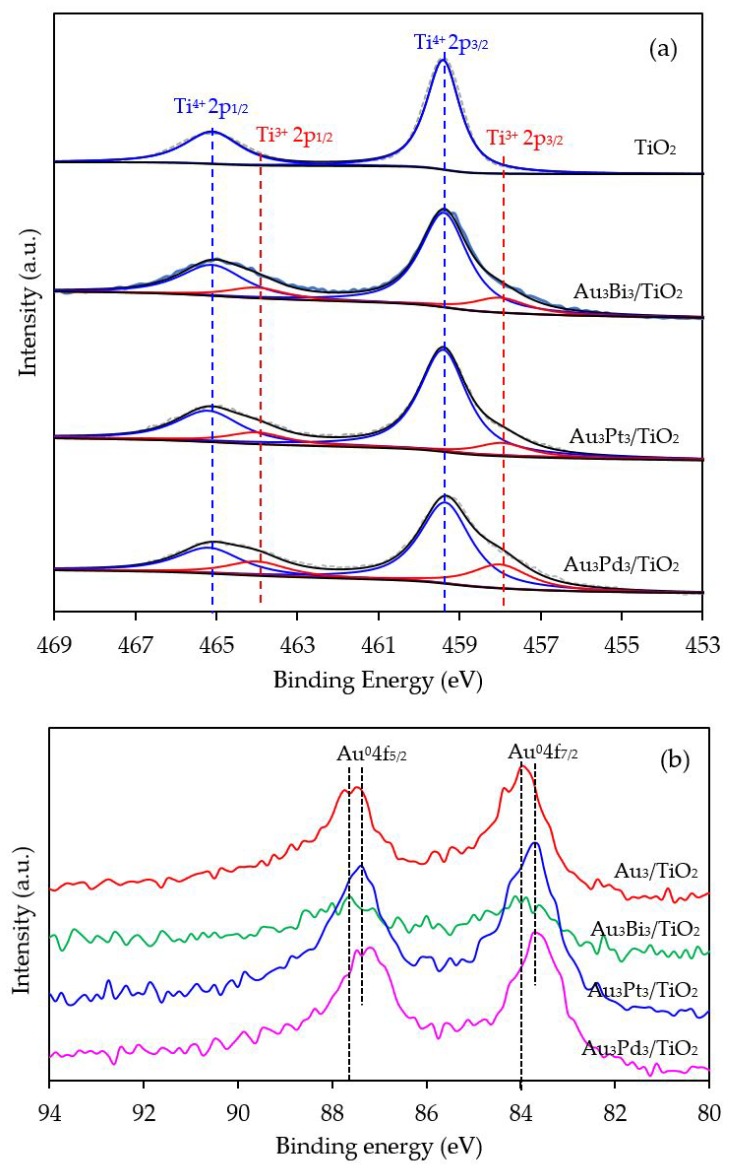
Representative HR-XPS spectra of (**a**) Ti2p and (**b**) Au4f of the TiO_2_, Au_3_/TiO_2_ and the different Au_3_M_3_/TiO_2_ photocatalysts.

To confirm the presence of the Ti^3+^ defective structures, Ti2p HR-XPS spectra of the Au_3_M_3_/TiO_2_ photocatalysts were investigated, and are shown in Figure 11a. The core-level Ti2p spectra still exhibited the two main symmetric peaks of Ti 2p_3/2_ and Ti 2p_1/2_ at a binding energy of 459.4 and 465.1 eV, respectively, with a difference of 5.7 eV, in accordance with the typical value of the Ti^4+^ state in TiO_2_. The shoulder peak of Ti2p at a low binding energy, assigned to the existence of the Ti^3+^ structure, was distinctly observed in all the Au_3_M_3_/TiO_2_ photocatalysts. The Ti^3+^/Ti^4+^ ratios, as obtained from the area under the deconvolution of both peaks, are listed in Table 1. The Au_3_M_3_/TiO_2_ photocatalysts all exhibited a higher Ti^3+^/Ti^4+^ ratio than the Au_3_/TiO_2_ and parent TiO_2_ photocatalysts, indicating that the addition of the bimetallic NPs (Au–Bi, Au–Pt and Au–Pd) induced a greater content of defective structures of TiO_2_ than the addition of the monometallic Au NPs. Among all the Au_3_M_3_/TiO_2_ photocatalysts, surprisingly, the Au_3_Pd_3_/TiO_2_ photocatalyst exhibited the highest Ti^3+^/Ti^4+^ ratio of 0.3561, which was 2.41-, 1.82- and 1.83-fold higher than that of the Au_3_/TiO_2_, Au_3_Bi_3_/TiO_2_ and Au_3_Pt_3_/TiO_2_ photocatalysts, respectively. The higher content of Ti^3+^ defect species in the bi- than mono-metallic system might be because the high content of decorated metal can incorporate into the TiO_2_ structure and induce formation of the Ti^3+^ defect site by transferring electrons between the decorated metal and oxygen vacancies of TiO_2_ in order to maintain the charge balance [73].

To distinguish the valence states of the decorated metal, the Au 4f level spectra of all Au_3_M_3_/TiO_2_ photocatalysts were analyzed, with respective examples shown in Figure 11b. The two main characteristic peaks of Au 4f_7/2_ and Au 4f_5/2_ were still observed with a spin-orbit splitting of 3.7 eV, indicating the presence of Au in the metallic state. A slight blue shift in the Au 4f binding energy was observed for the Au_3_Pt_3_/TiO_2_ and Au_3_Pd_3_/TiO_2_ photocatalysts in comparison with that of Au_3_/TiO_2_. Typically, the shift in the binding energy in each metal is attributed to various factors, such as a change in its chemical state or the charge compensation of added elements on the parent elements. However, in this case, the chemical state of Au in all the Au_3_M_3_/TiO_2_ photocatalysts was still in the metallic state (Figure 11b), and so this blue shift in the Au binding energy could be the result of charge compensation of the Pt or Pd elements on Au elements [74], by which Au accepted *sp*-electrons from Pt or Pd, but donated *d*-electrons to Pt or Pd, and possibly due to the partial formation of Au–M alloy structure, which are more negatively charged [75]. A low Au 4f intensity was observed in the case of the Au_3_Bi_3_/TiO_2_ photocatalyst compared with other two Au_3_M_3_/TiO_2_ photocatalysts, due to the presence of the Bi-wrapped Au NPs as a core–shell structure.

**Figure 12:**
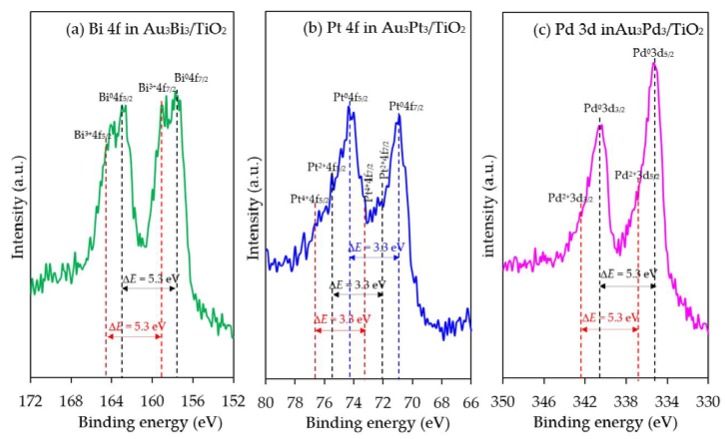
Representative HR-XPS spectra of Bi 4f, Pt 4f and Pd 3d in the respective Au_3_M_3_/TiO_2_ photocatalysts.

The chemical state of the second metal in all the photocatalysts was also analyzed by the HR-XPS. As demonstrated in Figure 12, all the second metals in the respective Au_3_M_3_/TiO_2_ photocatalysts were presented in both metallic and oxide forms. The peaks located at 162.9 eV and 157.6 eV for the Au_3_Bi_3_/TiO_2_ photocatalyst were assigned to Bi^0^ 4f_5/2_ and Bi^0^ 4f_7/2_, respectively, which is the characteristic value of the metallic Bi. A further two peaks at 164.5 eV and 159.2 eV were assigned to the Bi^3+^4f_5/2_ and Bi^3+^4f_7/2_ region, which are characteristic of Bi^3+^ in the as-prepared catalysts. These binding energy values of the Bi^3+^ ion-valent-form are too high to be assigned to the typical Bi^3+^ in bismuth oxide (Bi_2_O_3_), but rather were assigned to bismuth hydroxylated oxide (BiO(OH)) species on the photocatalyst surface [76]. For the Au_3_Pt_3_/TiO_2_ photocatalyst, the Pt 4f signals consisted of three pairs of doublets; the Pt^0^ 4f_5/2_ and Pt^0^ 4f_7/2_ peaks at binding energies of 74.2 eV and 70.9 eV, the Pt^2+^ 4f_5/2_ and Pt^2+^ 4f_7/2_ peaks at binding energies of 75.3 eV and 72.0 eV and the Pt^4+^ 4f_5/2_ and Pt^4+^ 4f_7/2_ peaks at binding energies of 76.5 eV and 73.2 eV, with an identical spin-orbit splitting of about 3.3 eV [77]. This suggests that the Pt NPs were present in both metallic zero-valent (Pt^0^) and metallic ion-valent (Pt^2+^ and Pt^4+^) forms. For the Au_3_Pd_3_/TiO_2_ photocatalyst, the most intensive doublet, at binding energies of 340.6 eV (Pd^0^ 3d_3/2_) and 335.3 eV (Pd^0^ 3d_5/2_), was attributed to metallic Pd. The shoulder peaks located at binding energies of around 342.2 eV and 336.9 eV were assigned to Pd^2+^ 3d_3/2_ and Pd^2+^ 3d_5/2_, respectively, which is characteristic of Pd^2+^ in PdO in the achieved catalyst. The spin-orbit splitting of about 5.3 eV in both the metallic Pd and Pd^2+^ was well in accordance with the reported value [14]. All the second metal NPs in the respective Au_3_M_3_/TiO_2_ catalysts existed predominantly in the metallic form, whereas the partial presence of the second metal in an oxide form might be attributed to the formation of a M–O bond driven by the oxygen chemisorption on the surface of the second metal nanostructure during the prepa ration process.

#### 3.2.2. Photocatalytic Activity

**Figure 13:**
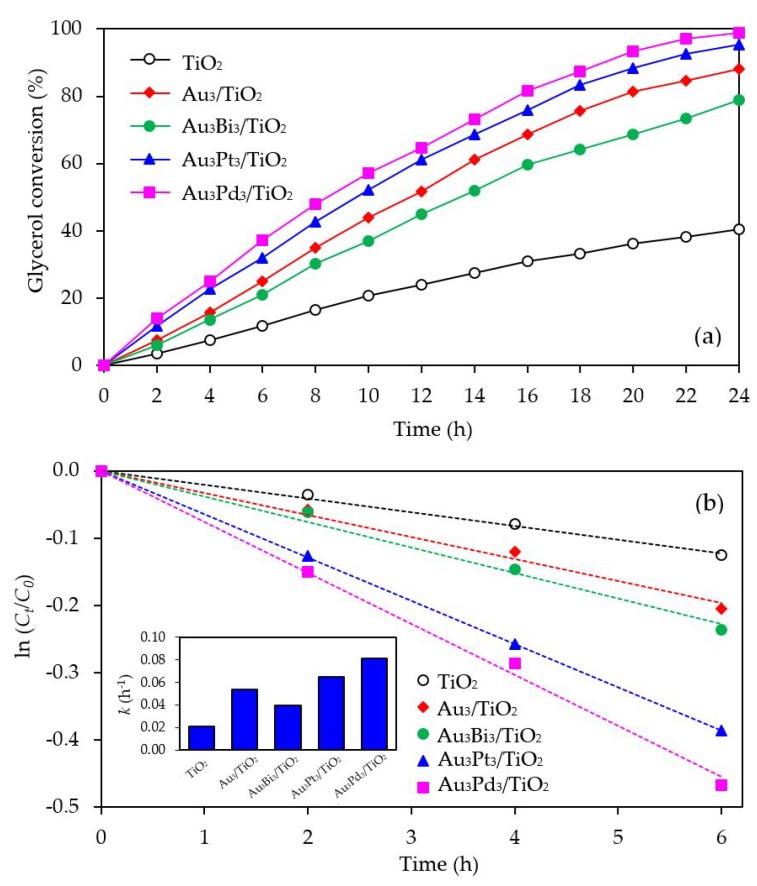
Representative (**a**) glycerol conversion and (**b**) a plot of ln (*C_t_*/*C*_0_) as a function of time in the presence of the respective Au_3_M_3_/TiO_2_ photocatalyst at a loading of 3.0 g/L, light intensity of 4.7 mW/cm^2^ and using O_2_ as the electron acceptor.

**Figure 14:**
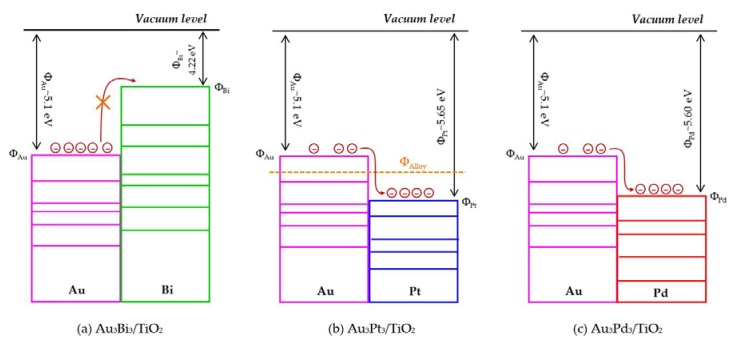
Schematic profiles of the contact metal with work function in the bimetallic NPs system.

The photocatalytic activity of the Au_3_M_3_/TiO_2_ photocatalysts was tested for the photocatalytic oxidation of glycerol to selected value-added compounds. As demonstrated in Figure 13a, the addition of Pt or Pd NPs on the Au_3_/TiO_2_ photocatalyst to form Au_3_Pt_3_/TiO_2_ and Au_3_Pd_3_/TiO_2_, respectively, enhanced the glycerol conversion, while the addition of Bi NPs retarded the reaction. The first order kinetic rate constants of glycerol conversion over Au_3_Bi_3_/TiO_2_, Au_3_Pt_3_/TiO_2_ and Au_3_Pd_3_/TiO_2_ were 0.0396, 0.0644 and 0.0810 h^−1^, respectively (Figure 13b). The Au_3_Bi_3_/TiO_2_ photocatalyst exhibited a lower photocatalytic activity than the Au_3_/TiO_2_ photocatalyst despite having a lower bandgap energy and higher defective structure, probably due to the core–shell structure of the AuBi NPs. That is, the LSPR behavior of the Au NPs was diminished when they were wrapped by the Bi species. Moreover, the decorated Bi NPs cannot extract the excited *e*^−^ from the Au or supported TiO_2_ due to its lower work function (4.22 eV) than that of Au (5.1 eV) and TiO_2_ (4.9 eV), resulting in unalleviated *e*^−^-*h*^+^ recombination (Figure 14a). The high photocatalytic activity of the Au_3_Bi_3_/TiO_2_ compared to the parent TiO_2_ was due to the presence of the valence band tail above the valence band, which was caused by the hybridization of the Bi 6s and O 2p orbital, and the existence of the Ti^3+^ state below the conduction band [53], which allowed an easy jump of photogenerated *e*^−^ from either the valence band tail level to either a shallow trap (Ti^3+^ state) or conduction band. In addition, as previously mentioned, the Bi6s is widely dispersed in the hybrid orbital of Bi6s-O2p leading to an increased charge mobility and consequently an improved photocatalytic activity of the parent photocatalyst [78]. 

For the Au_3_Pt_3_/TiO_2_ and Au_3_Pd_3_/TiO_2_ photocatalysts, the enhanced glycerol conversion compared with the Au_3_/TiO_2_ photocatalyst was probably due to their lower bandgap energy, which allowed the electron excitation at a lower photon energy and the presence of LSPR behavior in the decorated noble metal structure, resulting in the transfer of *e*^-^ from the metallic NPs to a shallow trap or conduction band of TiO_2_, or vice versa, under the light absorption. Moreover, in fact, the excited electron can be transferred by the driven work function. The difference in work function between Au and the second metal (Pt or Pd) can reduce the *e*^−^-*h^+^* recombination rate by transferring the *e*^-^ between the two metals after trapping the *e*^-^ from the conduction band of TiO_2_ into equilibrium [79]. That is, the work function of Au is 5.1 eV and those of Pt and Pd are 5.65 eV and 5.22 eV, respectively. The decorated Au–Pt NPs formed an alloy structure in the Au_3_Pt_3_/TiO_2_ photocatalyst, which can initiate a new Fermi level of alloy NPs somewhere between the Fermi level of Au and Pt (Figure 14b) and so could prolong the *e*^−^-*h*^+^ lifetime compared with the Au–Bi NPs. This was shorter than the two-stage transferring of an excited electron between the two individual Fermi levels of each decorated metal in the core–shell structure generated in the Au_3_Pd_3_/TiO_2_ photocatalyst (Figure 14c). This is the reason why the Au_3_Pd_3_/TiO_2_ exhibited a higher photocatalytic activity than the Au_3_Pt_3_/TiO_2_ photocatalyst.

The core–shell structure of the Au_3_Pd_3_/TiO_2_ photocatalyst did not exhibit a negative effect on the LSPR behavior in this photocatalyst, probably because the plasmon metallic state of Pd can still occur, leading to an easy transfer of *e*^-^ from the TiO_2_ conduction band to Pd NPs and/or Au NPs and vice versa. 

**Figure 15:**
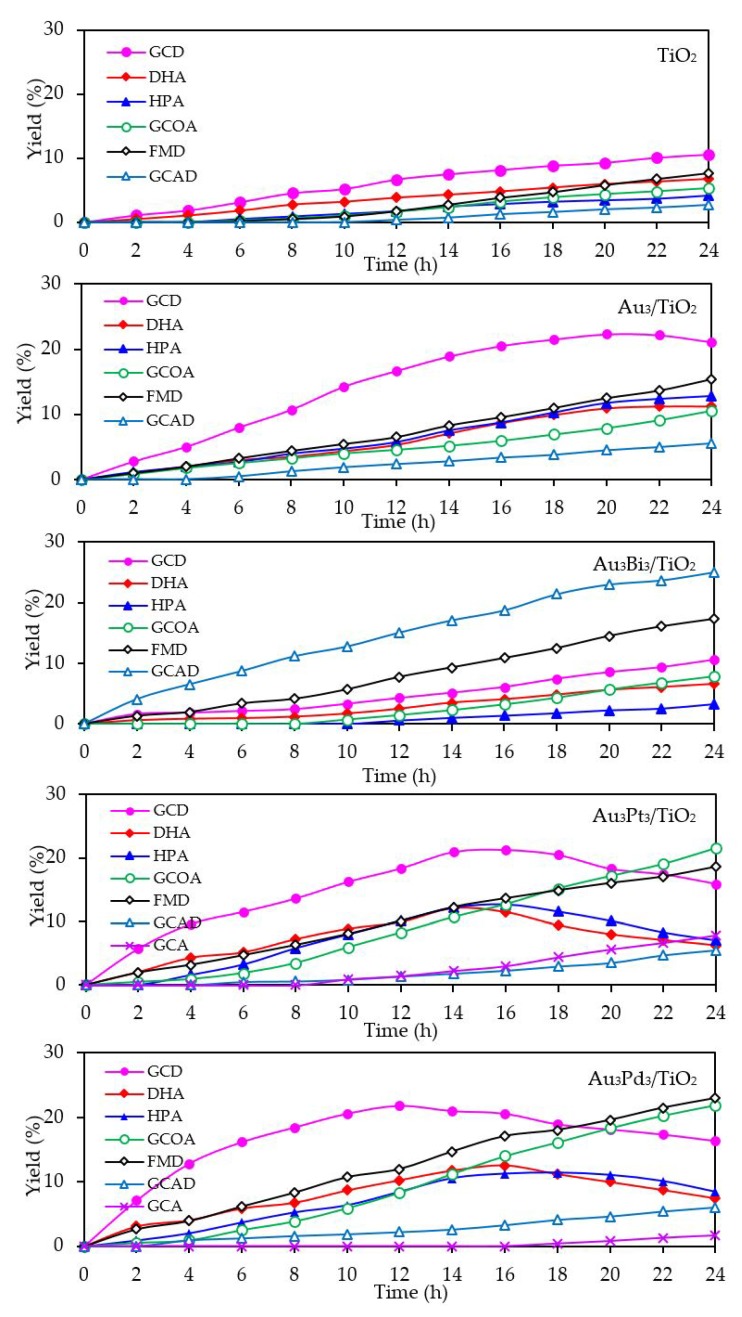
Yield of selected products obtained from the glycerol photocatalytic conversion via the TiO_2_, Au_3_/TiO_2_ and respective Au_3_M_3_/TiO_2_ photocatalysts at a loading of 3.0 g/L, light intensity of 4.7 mW/cm^2^, and using O_2_ as the electron acceptor.

With respect to the generated products, the same products (GCD, DHA, HPA, GCOA, FMD and GCAD) were generated via all the Au_3_M_3_/TiO_2_ photocatalysts and were similar to those generated in the presence of either the Au_3_/TiO_2_ or parent TiO_2_ photocatalyst (Figure 15). The principle product was GCD in the presence of all the evaluated photocatalysts except for Au_3_Bi_3_/TiO_2_, where GCAD was the principle product. As reported previously [53], monometallic Bi NPs decorated on TiO_2_ did not promote the formation of GCAD as a major product. Accordingly, it is suggested that the synergism between the bimetallic Au–Bi was a vital factor in the formation of GCAD from glycerol. Various published works on the aerobic oxidation of glycerol over bimetallic catalysts with Bi NPs have proposed that Bi played a major role as a geometric blocking site, which can facilitate the activation and transformation of the secondary hydroxyl group of glycerol [54,55,56]. In this present work, glycerol was mainly converted through the photocatalytic oxidation reaction over Au_3_Bi_3_/TiO_2_ to GCAD as the major product. It is hypothesized that the two terminal hydroxyl (-OH) groups of glycerol were bound on the surface of the Au–Bi NPs with Bi^3+^ species, whereas Bi adatoms function as blockers on the Au site, which is a high-energy site where the adsorption and reaction of the primary –OH group is preferred. This then serves to control the adsorbed orientation of glycerol molecules towards the preferential oxidation of the secondary –OH group to form DHA. In addition, due to the presence of various reactive oxygen species with a high oxidizing power in the photocatalytic system, the cleavage of the C–C bond of the generated DHA molecule to one molecule of GCAD and FMD is dramatically achieved. The generated FMD can eventually be oxidized to form CO_2_ as a gas-phase oxidation product. However, the synergetic role of Bi and the optimization of the Bi-promoted Au or other noble metals on TiO_2_ photocatalyst with the corresponding reaction conditions act to determine the highly selective oxidation of glycerol, which could also work to some extent that might be beneficial in academic research and the chemical industry.

## 4. Conclusions

Monometallic Au NPs with different metal contents and three types of Au-based bimetallic NPs (Au–Bi, Au–Pt and Au–Pd) with a nominal metal content of 3 wt. % were decorated on TiO_2_ by the sol-immobilization technique and then tested for photocatalytic oxidation of glycerol. The decoration of Au NPs in both mono- and bimetallic systems on TiO_2_ enhanced the photocatalytic glycerol conversion due to the narrowed bandgap energy and increased light harvesting capability of the as-prepared photocatalysts and the alleviation of *e*^−^-*h*^+^ recombination in the presence of the decorated metal. The Au_3_Pd_3_/TiO_2_ photocatalyst was found to be highly active and the most efficient tested photocatalyst for glycerol conversion, mainly because it had the lowest bandgap energy, the largest Ti^3+^ defect content and highest UV-Visible light absorption ability. The principle liquid phase product in the photocatalytic oxidation of glycerol over the Au*_x_*/TiO_2_, Au_3_Pt_3_/TiO_2_ and Au_3_Pd_3_/TiO_2_ photocatalysts was GCD. By contrast, Au_3_Bi_3_/TiO_2_ promoted the generation of GCAD as the major liquid phase product, which might be due to the promotional role of Bi species that act as a chelated site between the glycerol molecules and Au sites. This work paves a new way to undertake the rational design of mono- and bimetallic NPs decorated on TiO_2_ in order to enhance the performance of the photocatalytic process and promote the selective photocatalytic oxidation of glycerol. 

## Figures and Tables

**Table 1:** Properties of the as-synthesized Au*_x_*/TiO_2_ and Au_3_M_3_/TiO_2_ photocatalysts.

Photocatalyst	Au Loading (wt. %) ^a^	M Loading (wt. %) ^a^	Decorated Metal Particle Size (nm) ^b^	Bandgap Energy (eV) ^c^	Ti^3+^/Ti^4+ d^
Au_1_/TiO_2_	0.98 ± 0.10	n.d.	6.36 ± 0.75	3.00	0.1220
Au_3_/TiO_2_	3.02 ± 0.21	n.d.	6.69 ± 0.86	3.00	0.1478
Au_5_/TiO_2_	4.96 ± 0.19	n.d.	10.04 ± 1.11	2.94	0.2198
Au_7_/TiO_2_	7.08 ± 0.35	n.d.	10.84 ± 1.01	2.80	0.2412
Au_3_Bi_3_/TiO_2_	2.96 ± 0.12	3.11 ± 0.28	11.84 ± 1.11	2.85	0.1960
Au_3_Pt_3_/TiO_2_	3.06 ± 0.10	3.01 ± 0.20	7.61 ± 0.91	2.85	0.1941
Au_3_Pd_3_/TiO_2_	3.09 ± 0.14	3.05 ± 0.22	9.66 ± 1.06	2.79	0.3561

^a^ Estimated from the scanning electron microscopy–energy-dispersive X-ray spectrometry (SEM–EDS) analysis; ^b^ Average (± standard deviation (SD)) metal particle size obtained from the transmission electron microscopy (TEM) analysis; ^c^ Calculated from Tauc’s plot (plot of (*α*h*ν*)^1/*n*^ against (h*ν*)); ^d^ Calculated from area under deconvoluted high-resolution X-ray photoelectron spectroscopy (HR-XPS) peak of Ti2p; n.d., not detected.
